# Technical notes for digital polysomnography recording in sleep medicine practice

**DOI:** 10.4103/0972-2327.41887

**Published:** 2008

**Authors:** Manjari Tripathi

**Affiliations:** Department of Neurology, Associate Professor Neurology, AASM International Fellow 2008, Room number 705, Neurosciences Centre, AIIMS, Delhi, India

## Introduction

In our country, the standards of digital polysomnography (PSG) recordings vary widely, as is their interpretation. Proper recording and interpretation of digital PSG is crucial in the appropriate management of patients who undergo this test. This review is aimed to highlight the technical standards for digital PSG recording and scoring in sleep practice.

## Historical Background

The science of sleep medicine has evolved tremendously as a result of the development of tools that enable us to detect and document the activities of various physiological and pathological events that occur in the central nervous system accompanied by changes, which develop in the cardio respiratory, circulatory and autonomic nervous systems during sleep. This field is still evolving with tremendous pace in technology and interpretation. The initial steps towards this were taken as long back as 1875 when the ability to detect brain surface electrical activity in animals became available. In 1937, scalp brain recordings during PSG initially focused on visually identifiable patterns of brain activity during rapid eye movement (NREM) sleep, brain waveform patterns such as alpha and delta activities, and on well-isolated waveforms such as K complexes, spindles, vertex waves and posterior occipital sharp transients. In 1953, a landmark description of rapid eye movements (REMs) associated with respiratory and cardiac effects was given by Aserinsky and Kleitman[1] and later annotated as the REM stage of sleep. The first continuous sleep recording was performed in 1957 by Dement and Kleitman.[2] In as early as 1960, initial efforts for characterizing sleep patterns for performing an objective sleep scoring, which could permit inter-rater reliability in scoring, was started. A consensus meeting in April 1967, led to the formation of a standardized scoring manual; this manual was published from UCLA by Allan Rechtschaffen and Anthony Kales,[3,4] which we had followed till 2007. The existence of qualitative differences in sleep in newborns and children was identified; this resulted in publication of a separate manual for this age group in 1971.[5] In 2007, a more comprehensive scoring manual published by the American Academy of Sleep Medicine (AASM)[6] established the evolutionary and evidence-based changes that are occurring in this dynamic field. The various methods for evaluating sleep in the clinical laboratory are listed below:

- Overnight PSG
- Multiple sleep latency tests (MSLT)
- Maintenance of wakefulness tests (MWT)
- Video-PSG
- Extended electroencephalography (EEG) and PSG montage: In suspected seizure disorder
- Ambulatory PSG
- Actigraphy

### The indications for PSG are as follows:

- Sleep-related breathing disorders (obstructive sleep apneas, suspected upper-airway resistance syndrome, obesity hypoventilation syndrome, patient with neuromuscular disorder and breathing problems)
- CPAP-Bi-PAP titration
- For assessment of treatment efficacy after CPAP therapy
- After oral appliances or surgery
- Follow-up PSG if significant changes in symptoms occur
- Suspected narcolepsy (overnight PSG followed by MSLT)
- Parasomnias: unexplained nocturnal awakenings, unusual behavioral events in sleep-like sleep terror, REM behavior disorder or difference between seizure and a sleep disorder
- Periodic limb movements in sleep (PLMS)
- Insomnias (If suspected to have sleep apnea or PLMS, the cause is uncertain or failure of adequate behavioral and pharmacological treatment)
- MSLT will be required in suspected narcolepsy and unexplained daytime drowsiness
- MWT should be performed to detect response to continuous positive airway pressure (CPAP) therapy or medication
- A split study is performed when a previous scoring has been performed on the patients's sleep or if the clinician is reasonably certain that the patient could have severe OSA, and the first 2 h of sleep can document the sleep pattern with at least 20 apneas per hour. However, in many patients, REM may not occur during this time; hence, providing a false impression of the severity of hypoxia.

## Setting

It is mandatory that all persons involved in the recording of sleep use the most scientific and secure mechanisms. Maintaining a laboratory involves tremendous responsibility and having standard operating procedures (SOPs) manual, and technician's familiarity and continuous attendance with the procedure is very important.

The recording room should be sound proof, comfortable, have soothing soft colors on the wall; there should be a facility for video with lights switched off and an infrared video camera. The technician should be able to see from his station the patient's video and monitor continuously. Intervention is required if the physiological signals are missed due to problems with instrumentation or become obscured due to artifacts. The technician should also intervene if an acute change in the physiological status occurs and communicate these changes to the appropriate medical personnel on call. There should be an attached toilet in the room for the patients' comfort. The patient should be made to feel as comfortable as possible. The patient should be encouraged to bring along objects associated with sleep and all measures to help prevent the first night effect.

## Contraindications

There are no absolute contraindications to PSG when the indications are clearly established. However, risk-benefit ratios should be assessed if medically unstable inpatients are to be transferred from the clinical setting to a sleep laboratory for overnight PSG; all resuscitative equipment should be made available in the room.

## Precautions/Complications

Skin irritation may occur as a result of the adhesive used to attach electrodes to the patient. At the conclusion of the study, adhesive remover is used to dissolve the adhesive on the patients' skin. Care should be taken to prevent the use of the adhesive to attach the EOG around the eyes. Due to the high flammability of collodion and acetone, they should be used with caution, especially in those patients who require supplemental oxygen. Patients with parasomnias or seizures may be at risk for injury related to movements during sleep. The integrity of PSG equipment's electrical isolation should be certified by engineering or biomedical personnel qualified to make such assessment. Institution-specific policies, protective measures and guidelines describing personnel responsibilities and appropriate responses in such situations should be developed.

## Assessment of test quality

With respect to sleep-related respiratory disturbances, PSG should either confirm or eliminate a diagnosis. Reporting should be performed following a standard format; automated scoring should be strongly discouraged because of the false over or under scoring. All recordings should be manually scored. Documentation of findings suggested therapeutic intervention, and/or other clinical decisions resulting from PSG should be noted in the patients' chart. Each laboratory should devise and implement indicators of quality assurance with respect to equipment calibration and maintenance, patient preparation and monitoring, scoring methodology, and inter-rater scoring issues.

## Monitoring

Patients should have been previously examined by the physician in the clinic, explained and consented for the procedure. They should be requested to avoid stimulants before the procedure. Ideally, two weeks prior to the procedure, the medication should be tapered and stopped. If MSLT is being performed, it should be in the morning after the PSG. The Epworth sleep scale should be recorded and a detailed history of the patients complaints and habitual sleep pattern be recorded. The reason and indication for performing the study should be clearly documented. A complete physical examination such as height, weight, BMI, perioral characteristics of the patient should be documented. The type of mask to be selected during the CPAP titration will depend not only on the patient's preferences and affordability but also on his breathing patterns and predominant sleep postures.

## Calibration

**Figure 1:**
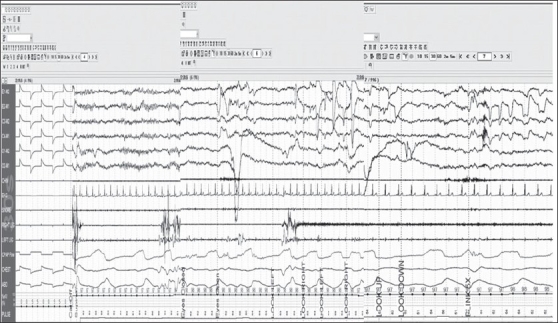
Calibration 30-s epoch

Calibration should be performed both at the beginning and the end of the study under the following categories. Physiological/biological calibration has to be performed as follows: ask the patient to keep the eyes open, look straight ahead for 30 s, close the eyes, look straight ahead for 30 s, look to the left and right, repeat up and down, hold head still, blink eyes slowly five times, grit teeth, clench jaw or smile, inhale and exhale, hold breath for 10 s, flex right foot, flex left foot, flex right hand, flex left hand, turn the patient to either side to check on the body position sensors. Mechanical calibration should also be performed [Figure 1].

The patient variables to be monitored during PSG as recommended by AASM guidelines, 2007, are listed below.

### Recording parameters

1. EEG derivations
2. Electro-oculogram (EOG) derivations
3. Chin electromyography (EMG)
4. Leg EMG derivations
5. Airflow parameters
6. Effort parameters
7. Oxygen saturation
8. Body position
9. Electrocardiography (ECG)

1) **EEG:** EEG electrode position is determined by international 10–20 System.[7] A minimum of three EEG derivations are recommended to sample the activity from the frontal (F), central (C) and occipital regions (O).

The recommended derivations are:

- F_4_-M_1_
- C_4_-M_1_
- O_2_-M_1_

Backup electrodes should be placed at F_3_, C_3_, O_1_ referenced to M_2_ to allow display of F_3_-M_2_, C_3_-M_2_ and O_1_-M_2_ if electrodes malfunction during the study.

Alternative acceptable derivations are:

- Fz-Cz
- Cz-Oz
- C_4_-M_1_

Backup electrodes should be placed at Fpz, C_3_, O_1_, and M_2_ to enable substitution of Fpz for Fz, C_3_ for Cz or C_4_, O_1_ for O_2_ and M_2_ for M_1_ if electrodes malfunction during the study.

2) **EOG:** The recommended EOG derivations are:

- E_1_-M_2_ (E_1_ is placed 1 cm below the left outer canthus)
- E_2_-M_2_ (E_2_ is placed 1 cm above the right outer canthus)

Alternative acceptable derivations are:

- E_1_-FPz (E_1_ is placed 1 cm below and 1 cm lateral to the outer of the left eye)
- E_2_-Fpz (E_2_ is placed 1 cm below and 1 cm lateral to the canthus of the right eye)

3) **EMG:** Three electrodes should be placed to record chin EMG:

- One in the midline 1 cm above the inferior edge of the mandible
- One 2 cm below the inferior edge of the mandible and 2 cm to the right of the midline
- One 2 cm below the inferior edge of the mandible and 2 cm to the left of the midline

**Figure 2:**
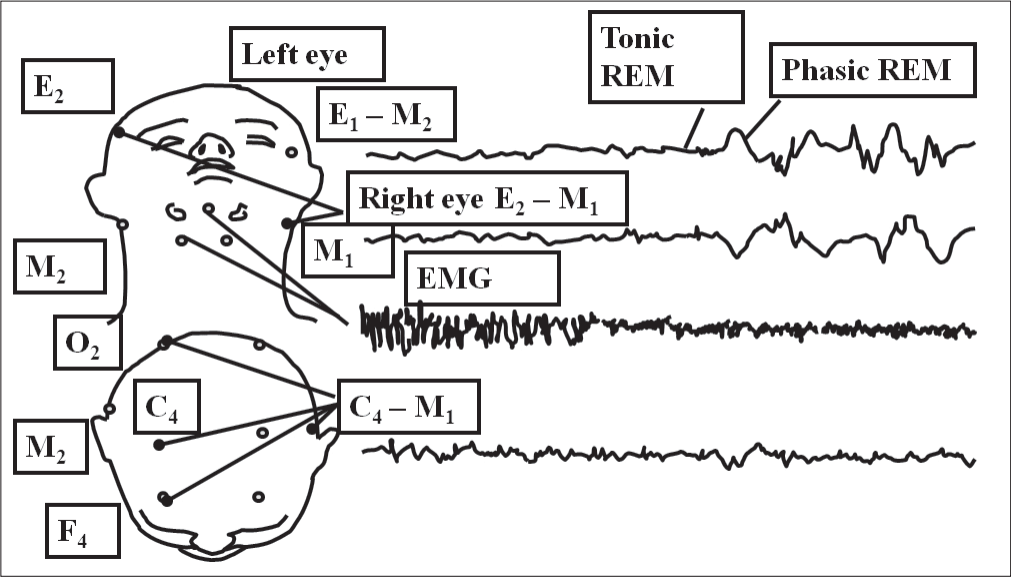
Electrode placement {(EEG 10 × 20), CHIN, EOG}

The standard chin EMG derivation consists of either of the electrodes below the mandible referred to the electrode above the mandible. The other inferior electrode is a backup electrode to permit continuous display of EMG activity if one of the primary electrodes malfunctions [Figure 2].

**Figure 3:**
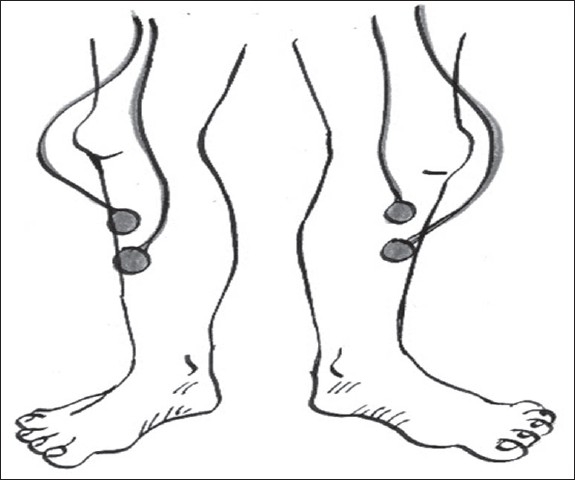
EMG tibialis anterior

4) **Scoring of leg movements:** Surface electrodes should be placed longitudinally and symmetrically around the middle of the muscle so that they are 2–3 cm apart [Figure 3]. Both legs can be used for monitoring the movements, especially if alternate leg muscle activation is suspected (ALMA). The upper limbs may also be sampled, especially if they are the ones involved in severe PLMS or RBD.

5) **Airflow parameters:** The current guidelines recommend the use of a thermal sensor, which is placed in the patient's nostril to detect the apnea; the nasal pressure transducer is used for identifying hypopnea. Ideally, both the sensor and transducer should be used.

6) **Effort parameters:** Respiratory inductance plethysmography (RIP belts), individual chest and abdominal belts with peizo electrodes may be used for detecting respiratory effort. Esophageal manometry is difficult to perform and is uncomfortable for the patient who already has a difficulty in sleeping.

7) **Oxygen saturation:** Pulse oximetry with a maximum acceptable signal averaging time of 3 s should be used for detection of blood oxygen.

8) **Body position:** Body position sensors may be unreliable, and a video film of the patient should be acquired to confirm the position when there is any suspicion.

9) **ECG:** A single modified electrocardiograph Lead II, which uses torso electrode placement, is recommended.

## Sleep Scoring

Data to be recorded in the study is as given below. All the following steps are recommended.

### A. Sleep scoring data

1. Lights out clock time (h: min)
2. Lights on clock time (h: min)
3. Total sleep time (TST; min)
4. Total recording time (“lights out” to “lights on” in min)
5. Sleep latency (SL; lights out to the first epoch of any sleep in min)
6. Stage R latency (sleep onset to the first epoch of stage R in min)
7. Wake after sleep onset (WASO; stage W during A4, minus A5, in min)
8. Percent sleep efficiency (A3/A4) ×100
9. Time in each stage (min)
10. Percentage of TST in each stage (A9 values/A3) ×100

### B. Arousal events

1. The number of arousals
2. The arousal index (ArI; B1×60/TST)

### C. Respiratory events

1. Number of obstructive apneas
2. Number of mixed apneas
3. Number of central apneas
4. Number of hypopneas
5. Number of apneas + hypopneas
6. Apnea index (AI; (C1+C2+C3)×60/TST)
7. Hyponea index (AHI; C4×60/TST)
8. Apnea+ Hypopneas index (AHI; C5×60/TST)
9. Respiratory effort-related arousals (RERAS), total number
10. Respiratory effort-related arousals index, (C9×60/TST)
11. Oxygen desaturations ≥3% or ≥4% - total number
12. Oxygen desaturations index ≥3% or ≥4 %(DI; C11×60/TST) [9–12 are optional]
13. Continuous oxygen saturation, mean value
14. Minimum oxygen saturation during sleep
15. Occurrence of hypoventilation (yes/no)
16. Occurrence of Cheyne Stokes breathing (yes/no)

### D. Cardiac events

The average heart rate (HR) during sleep, highest HR during sleep and also the entire recording should be noted. Occurrence of the following arrythmias should be noted with the duration of pause. Bradycardia- note the lowest HR, asystole – report the longest pause, sinus tachycardia- report the highest rate, narrow complex tachycardia- report the highest rate, wide complex tachycardia- report the highest rate and the occurrence of atrial fibrillation.

### E. Movement events

The number of PLMS should be noted; PLMS with arousals (PLMSAr), PLMS index (PLMSI; PLMS × 60/TST) and PLMS arousal index (PLMSIArl; PLMSAr × 60/TST) should also be noted.

The summary of the report should have the following headings:

Findings related to sleep diagnosis
EEG abnormalities
ECG abnormalities
Behavioral observations
Hypnogram (optional)

### Digital Specifications for Routine PSG Recording

Maximum Electrode Impedances5 KΩMinimum Digital Resolution12 bits per sample

**Sampling Rates****Desirable****Minimal**EEG500 Hz200 HzEOG500 Hz200 HzEMG500 Hz200 HzECG500 Hz200 HzAirflow100 Hz25 HzOximetry25 Hz10 HzNasal pressure100 Hz25 HzEsophageal pressure100 Hz25 HzBody position1 Hz1 HzSnoring sounds500 Hz200 HzRib cage and abdominal moments100 Hz25 Hz

**Routinely Recorded Filter Settings****Low Frequency Filter****High Frequency Filter**EEG0.3 Hz35 HzEOG0.3 Hz35 HzEMG10 Hz100 HzECG0.3 Hz70 HzRespiration0.1 Hz15 HzSnoring10 Hz100 Hz

### The digital recording system must include the following features:

1. A toggle switch-permitting visual (onscreen) standard negative 50-µv DC calibration signal for all channels to demonstrate polarity, amplitude and time constant settings for each recorded parameters.
2. A separate 50/60 Hz filter control for each
3. The capability of selecting sampling rates for each channel
4. A method of measuring actual individual electrode impedance against a reference (the latter may be the sum of all other applied electrodes)
5. The capability of retaining and viewing the data in the same manner in which it was recorded by the attending technologist (i.e., retain and display all derivation changes, sensitivity adjustments, filter settings and temporal resolution)
6. The capability of retrieving and viewing the data in the same manner as it appeared when it was scored by the scoring technologist.
7. A filter design for data collection, which functionally simulates or replicates conventional (analog style) frequency response curves rather than removing all activity and harmonics within the specified bandwidth.

### Rules for PSG Display and Display Manipulation

1. Resolution of digital screen and video card should be at least 1600 × 1200 for display and scoring of raw PSG data
2. Histogram with stage, respiratory events, leg movement events, O_2_ saturation and arousals, with cursor positioning on histogram and ability to jump to the page of selection.
3. Ability to view a screen on a scale ranging from the entire night to windows as small as 5 s.
4. Recorded video data should be synchronized with PSG data and have an accuracy of at least one video frame per second.
5. Automatic page turning and scrolling, channel-off control key or toggle, channel invert control key or toggle, ability to change the order of channel by click and drag, display setup profiles along with colors that may be activated at any time, Fast Fourier Transformation or spectral analysis on specifiable intervals is optional.

Whether sleep stage scoring was performed should be identified for all systems. The capability to turn off and on, as demanded, highlighting for patterns identifying sleep stage decisions (for example, sleep spindle complex, alpha and delta), patterns identifying the respiratory analysis (for example, apneas, hyponeas and desaturations), patterns identifying movement analysis (for example, PLMs) and automatic calculation of the time in the highlighted area are optional.

## Scoring of sleep stages

### A. Stages of sleep

1. The following terminologies are recommended for the stages of sleep:
  - Stage W (Wakefulness)
  - Stage N1 (NREM 1)
  - Stage N2 (NREM 2)
  - Stage N3 (NREM 3 and 4 in R and K, respectively)
  - Stage R (REM)

### B. Scoring sleep by epochs

Score sleep stages in 30 s sequential epochs commencing from the beginning of the study, and assign a stage to each epoch. If 2 or more stages coexist during a single epoch, assign to the stage comprising the largest portion of the epoch.

Stage W is defined by the presence of an alpha rhythm: these are trains of sinusoidal 8–13 Hz activity over the occipital regions and are best seen with eyes closed and attenuated with eye opening. Eye blinks appear as conjugate eye movements consisting of 0.5–2 Hz present in wakefulness with eyes open or closed.

Reading eye movements may be seen as trains of conjugate eye movements consisting of a phase followed by a rapid phase in the opposite directions as the subjects read. REM that may also be seen are conjugate, irregular and sharply peaked eye movements. They may be seen in the awake state when the patient scans the environment.

Rules

Score epochs as stage W when more than 50% of the alpha rhythm is over the occipital region.

**Figure 4:**
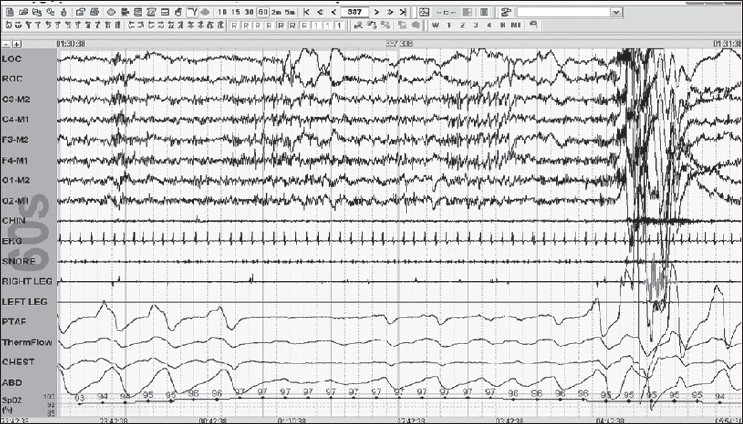
Stage W: note the eye movements with high chin tone, 30-s epoch

Score epochs without visually discernable alpha rhythm as stage W if any of the following are present [Figure 4]:

1. Eye blinks at a frequency of 0.5–2 Hz
2. Reading eye movements
3. Irregular conjugate REMs associated with normal or high chin muscle tone

**Figure 5:**
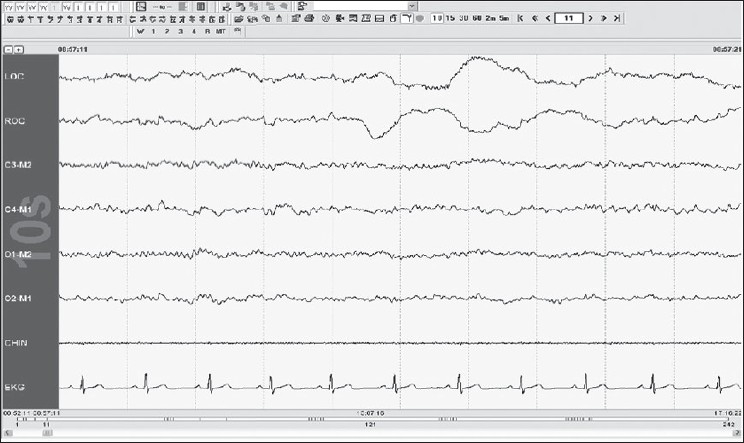
Stage N1: SEMs are seen with gradual drop out of alpha, 10-s epoch

Stage N1 is defined by the presence of slow eye movements (SEM) Conjugate, reasonably regular and sinusoidal eye movements with an initial deflection usually lasting >500 ms [Figure 5]. The EEG is low amplitude, mixed frequency activity, predominantly of 4–7 Hz. Presence of vertex sharp waves (V waves): are sharply contoured waves with duration <0.5 s seen mostly over the central region and are distinguishable from the background activity. Sleep onset is defined as the beginning of the first epoch scored as any other than stage W (In most subjects, this will usually be the first epoch of stage N1).

Rules

- In subjects who generate alpha rhythm, score stage N1 if alpha rhythm is attenuated and replaced by low amplitude, mixed frequency activity for more than 50% of the epoch.
- In subjects who do not generate alpha rhythm, score stage N1 commencing with the earliest of any of the following phenomena.
  1. Activity in the range of 4–7 Hz with slowing of background frequencies by ≥1 Hz from those of stage W.
  2. Vertex sharp waves.
  3. Slow eye movements (SEM).

**Figure 6:**
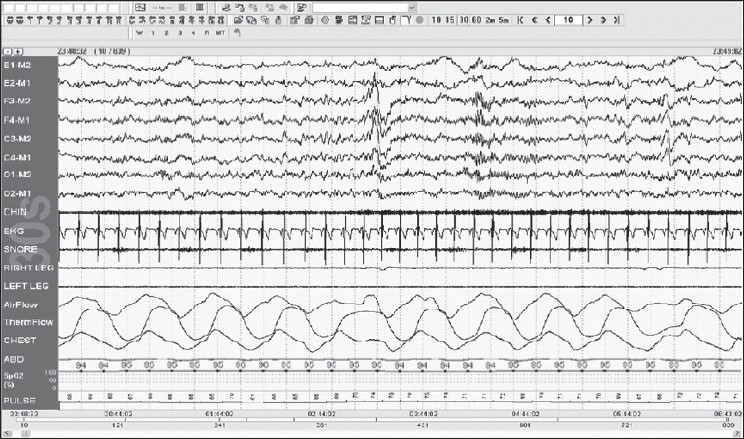
Stage N2: K complexes and sleep spindles seen, 30-s epoch

Stage N2: Is defined by the appearance of K complexes, which are well-delineated negative sharp waves immediately followed by a positive component standing out from the background EEG, with total duration ≥0.5 s, usually maximal in amplitude when recorded using frontal derivations. For an arousal to be associated with a K complex, it should commence no more than 1 s after the termination of the K complex. Sleep Spindle are present in N2; these are trains of distinct waves with frequency pattern of 11–16 Hz (most commonly 12–14 Hz) with a duration ≥0.5 s, usually maximal in amplitude in the central derivations [Figure 6].

Rules

- The following rule defines that the beginning of the period of stage N2 Sleep:
  1. Begin scoring stage N2 (in absence of criteria for N3) if one or both of the following occur during the first half of this epoch or the last half of the pervious epoch.
    - One or more K complexes unassociated with arousals.
    - One or more trains of sleep spindles.
- The following rule defines the continuation of the period of stage N2 Sleep.
  1. Continue to score epochs with low amplitude, mixed frequency EEG activity without K complex or sleep spindles as stage N2 if they are preceded by (a) K complexes unassociated with arousals or (b) sleep spindles
  2. End stage N2 sleep when either of the following events occurs:
    - Transition to stage W
    - An arousal (change to stage N1 until a K complex unassociated with an arousal or a sleep spindle occurs)
    - A major body movement followed by SEM with low amplitude mixed frequency EEG without nonarousal-associated K complexes or sleep spindles (score the epoch following the major movements as stage N1; score the epoch as stage N2 if there are no SEMs)
    - Transition to stage N3
    - Transition to stage R

**Figure 7:**
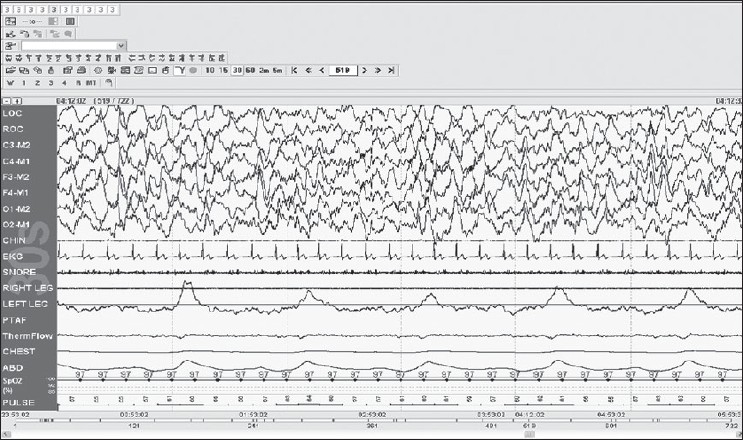
Stage N3: Delta slow waves are seen, 30-s epoch

Stage N3: is defined by slow wave activity, which are waves of frequency 0.5–2 Hz with a peak-to-peak amplitude >75 μv, as measured over the frontal regions [Figure 7].

Rule

Score stage N3 when 20% or more of an epoch consists of slow wave activity irrespective of age. Spindles may persist in N3; alpha intrusions and alpha delta may also be seen, and eye movements are typically absent.

**Figure 8:**
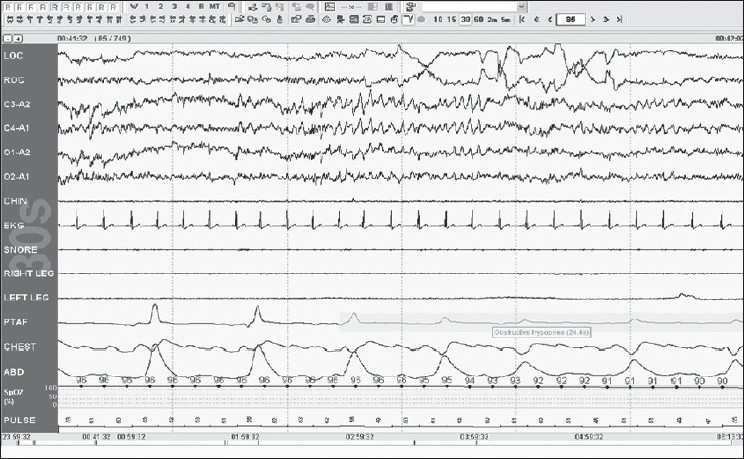
Stage R: Note saw tooth waves with REMS; further, note that the criteria are fulfilled for obstructive hypopnea 30-s epoch

Stage R is defined by REMs; these are conjugate, irregular and sharply peaked eye movements with initial defection usually lasting <500 ms. Low Chin EMG Tone is the hallmark of this stage; the baseline EMG activity in chin derivation is not higher than in any sleep stage and is usually at the lowest level of the entire recording. Saw tooth waves are seen; these are trains of sharply contoured or triangular, often serrated [Figure 8] waves of 2–6 Hz with maximal amplitude over the central head regions and often, but not always, is preceded by a burst of REMs.

Transient muscle activity: Short irregular bursts of EMG activity usually of duration <0.25 s superimposed on low EMG tone can be seen. The activity may be seen in the chin or anterior tibial EMG derivations as well as EEG or EOG derivations, the latter indicating activity of cranial nerve innervated muscles. The activity is maximal in association with the REMs.

Rules

- Score stage R sleep for epochs with all the following phenomena:
  - Low amplitude, mixed frequency EEG
  - Low Chin EMG tone
  - REMs
- The following rule defines the continuation of a period state R sleep:
  Continue to score R, even in the absence of REMs, for epochs following one or more epochs of stage R as defined above, if EEG continues to show low amplitude, mixed frequency activity without K complexes or sleep spindles and the chin EMG tone remains low.
- The following rule defines the end of a period stage R sleep:
  Stop scoring stage R sleep when one or more of the following occur:
  There is a transition to stage W or N3
  - An increase in chin EMG tone above the level of stage R is seen and criteria for stage N1 are fulfilled
  - An arousal occurs followed by low amplitude, mixed frequency EEG and SEM (score as stage N1; if no SEMs and chin EMG tone remains, continue to score as stage R)
  - A major body movement followed by SEMs and low amplitude mixed frequency EEG without nonarousal-associated K complexes or sleep spindles (score the epoch following body movement as stage N1; if no SEMs are seen and EMG tone remains low, continue to score as stage R)
  - If one or more nonarousal-associated K complexes or sleep spindles are present in the first half of the epoch in the absence of REMs, even if chin EMG tone remains low, score as stage N2.
- Score epochs at the transition between stages N2 and R as follows:
  1. In between epochs of definite stages N2 and R, score an epoch with a distinct drop in chin EMG in the first half of the epoch to the level seen in stage R as stage R if all the following criteria are fulfilled, even in the absence of REMs
    - Presence of non-arousal associated K complexes or sleep spindles
    - Absence of rapid eye movements.
  2. In between epochs of definite stages N2 and R, score an epoch with a distinct drop in chin EMG in the first half of the epoch to the level seen in stage R as stage N2 if all of the following criteria are fulfilled.
    - Absence of nonarousal-associated K complexes
    - Absence of or sleep spindles.
  3. In between epochs of definite stage N2 with minimal chin EMG tone and definite stage R without further drop in chin EMG tone, score epochs as stage R if all of the following criteria are fulfilled, even in the absence of REMs that there is a) Absence of nonarousal-associated K complexes b) Absence of sleep spindles.

## Major Body Movements

**Figure 9:**
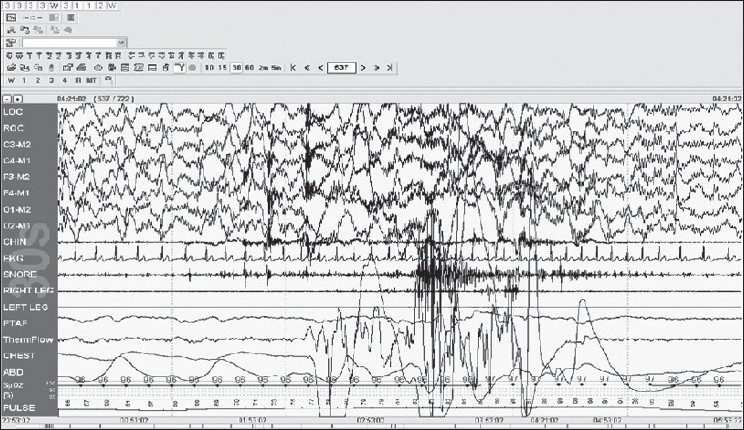
Major body movement occupying more than half of the epoch and obscuring the basal rhythm 30-s epoch

Definition of Major body movement: Movement and muscle artifact that obscure the EEG for more than half an epoch to the extent that the sleep stage cannot be determined [Figure 9]

Rules: Score a epoch with a major body movement as follows:

- If alpha rhythm is present for part of the epoch (even <15 s duration), score stage W
- If no alpha rhythm is discernable, but an epoch scorable as stage W either precedes or follows the epoch with a major body movement, score as stage W.
- Otherwise, score the epoch as the one that follows it.

**Figure 10:**
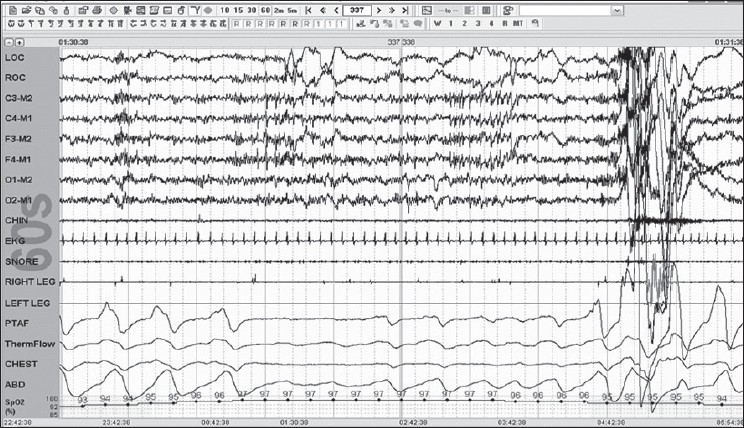
Arousal in REM note the increase in chin tone, which is essential for scoring an REM arousal associated is a period of mixed apnea 60-s epoch

Arousal rule: Score an arousal during any sleep stage if there is an abrupt shift in EEG frequency in any range (but not spindles); this shift should last for 3 s with at least 10 s of stable sleep preceding the change. Scoring of an arousal during REM requires an increase in chin EMG lasting for at least 1 s [Figure 10].

Scoring PLMS: The duration of a significant leg movement should be between 0.5–10 s. The maximum increase in the amplitude should be 8 μV in the EMG voltage above the resting EMG. A PLM series should have at least 4 LMs in a series. The minimum duration between these should be 5 ms, and the maximum duration, 90 ms. If both legs move and the separation between them is less than 5 s then they are scored as one movement.

Scoring Apneas: The amplitude criteria for scoring an apnea are at least a 90% drop or more in the thermal sensor excursion, lasting for at least 10 s. It should be labeled as obstructive if the efforts (respiratory and abdominal continue) are seen; it should be called central if none of these excursions are seen, and mixed [Figure 10], if this effort is resumed toward the end of the period of apnea.

Scoring Hypopnea: The duration of hypopnea should be at least 10 s. The drop in the amplitude of the nasal transducer is >30%, with a 4% drop in saturation [Figure 8] or >50%, with a 3% drop in the saturation.

**Figure 11:**
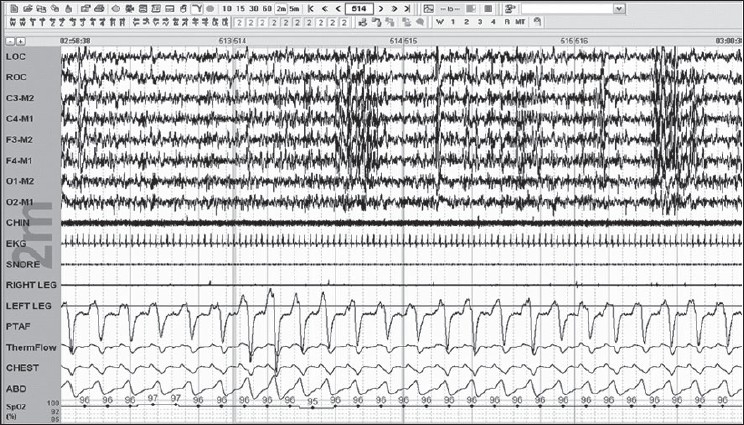
RERAs/UARS: multiple arousals in 2-m epoch; this did not fulfill the 30% or 50% drop criteria 2-m epoch

Scoring respiratory effort-related arousals: The duration of change should be more than 10 s seen mainly as a flattening or deformed nasal pressure signal accompanied by an arousal from sleep which does not fulfill the criteria of apnea or hypopnea [Figure 11].

Hypoventilation should be suspected if there is a >10 mmHg increase in PaCO_2_ by either end tidal or transcutaneous CO_2_ measures. Cheyne stokes pattern should be scored if there are at least 3 consecutive cycles of cyclical crescendo and decrescendo change in breathing that lasts for 10 min continuously, with 5 or more apneas or hypopneas per hour.

**MSLT:** Each laboratory should have a set protocol for both MSLT and MWT, although MSLT is performed more often. MSLT comprises 5 naps performed at least 2 h after the end of the PSG. It should be performed in a comfortable, soundproof, dark bedroom, while wearing street clothes. The patient is instructed to fall asleep; he should be requested to avoid smoking or caffeine between naps, and he can read a book in between naps. Each nap time is approximately 20 min with 2 h between each nap. It records the latency for each nap (time between light-out and sleep onset); the mean sleep latency can then be calculated. Latency below 5 min is abnormal and suggestive of severe sleepiness, 5–10 min is moderate and 10–15 min is mild sleepiness. The presence of two or more sleep onset REMs (SOREMS- REM onset within 15 min of sleeping) is suggestive of narcolepsy; however, sleep apnea and acute withdrawal from REM suppressing medications can also cause this.

**MWT:** It tests the patient's ability to remain awake and the instruction provided to the patient is to stay awake in a comfortable sitting position in a dark room for five 20-min trials. The normal sleep latency should be 11 min. The test may be helpful in specific pharmacological trials and also in monitoring efficacy to treatment.

The continuous 24- or 36-h ambulatory PSG provides information on the number, duration, times and types of daytime sleep episodes, as well as documenting nighttime sleep disruptions. In addition, this long PSG recording may identify the dissociated REM sleep-inhibitory process characterizing cataplexy by showing the elimination of chin and muscle twitches that are typical of REM sleep.

**Actigraphy:** It is reliable and valid for detecting sleep in healthy populations but less reliable for assessing disturbed sleep. It is a useful adjunct to the routine evaluation of insomnia, sleep state misperception, circadian rhythm disorders and excessive sleepiness, and it helps in the diagnosis of PLMS and restless legs syndrome. It is also useful in specific populations affected with dementia, such as children or older adults.

The rules differ slightly in children, the description of which is beyond the preview of this report. The author recommends reading of the 2007 AASM manual for the same.

Since reading and interpreting sleep studies involves eyeballing, reading more and more studies will enhance the visual pattern recognition memory and register the findings, hence increasing the scorer's confidence, which is strongly recommended. Subjective evaluation of the procedure by the patient should be performed for quality control and also for knowing the efficacy of the CPAP therapy.

## Frequency

A second PSG study may be indicated under the following conditions. If the first study is technically inadequate due to equipment failure; if the subject could not sleep or slept for an insufficient amount of time due to first night effect to allow a clinical diagnosis; if initiation of therapy or confirmation of the efficacy of prescribed therapy is needed.

## Infection control

Practitioners should exercise universal precautions and precautions for the prevention of the spread of infections. Nondisposable items for patients' use (e.g., pneumotachometers, face masks, electrodes) should undergo cleaning and sterilization procedures as recommended by the manufacturer. If sterilization is not feasible, high-level disinfection is warranted. The syringe and flat-tipped needle used for injecting transduction gel into the EEG, EOG and EMG electrodes should be discarded after use. With respect to body-position sensors, inductance and impedance pneumography, abdominal or thoracic strain gauges or piezoelectric belts, no specialized precautions are generally required. Gas sterilization may be employed if the sensors or belts become contaminated with body fluids. Some thermistors are equipped with disposable sensors. If nondisposable sensors are used, they should be cleaned and subjected to high-level disinfection after use.

**Portable PSG:** The American sleep disorders association has divided portable monitoring into four types. Type I: Attended polysomnography, Type II- Unattended polysomnography, Type III- Modified portable sleep apnea testing (min 4 channels), Type IV- Continuous single or dual bioparameter recording. The problems with the portable recordings are the artifacts and loss of data.

**Pitfalls of PSG:** There is no standardized protocol used in all laboratories in the country as yet; it is labor intensive, time consuming and expensive. A single night's PSG may miss the diagnosis of mild OSAS, PLMS, parasomnias and nocturnal seizures and other episodic event. PSG data and patient's clinical history may not be concordant; PSG may be confounded by the first night effect; and the standard PSG does not measure PaCO_2_ and thus may miss hypoventilation.

Exchange of clinical PSG: Portability of the record is crucial and it should be possible to perform the same with the current digital systems available.

## Conclusion

The sleep neurologist and the technologist who is the most important person responsible for obtaining a good recording should take into account the safety and well-being of patient first, should be trained and skilled, ensuring a high quality recording, be able to detect and correct artifacts (not covered in this review), record all abnormal events and be alert for the possibility of the patient attempting to get out of bed, detect and initiate management of medical emergencies and be well versed with the functioning of CPAP, BiPAP and other ventilatory devices.

This technical report states all recommended rules with optional or alternative rules being specifically mentioned as well.
